# Interaction of plant growth regulators and reactive oxygen species to regulate petal senescence in wallflowers (*Erysimum linifolium*)

**DOI:** 10.1186/s12870-016-0766-8

**Published:** 2016-04-02

**Authors:** Faezah Mohd Salleh, Lorenzo Mariotti, Natasha D. Spadafora, Anna M. Price, Piero Picciarelli, Carol Wagstaff, Lara Lombardi, Hilary Rogers

**Affiliations:** School of Biosciences, Cardiff University, Main Building, Park Place, Cardiff, CF10 3TL UK; Current address: Faculty of Biosciences and Medical Engineering, Universiti Teknologi Malaysia, 81310 Johor Bahru, Johor Malaysia; Current address: Queen Mary University of London, Mile End Road, London, E1 4NS UK; Department of Biology, University of Pisa, Via Ghini 5, 56126 Pisa, Italy; Department of Agriculture, Food and Environment, University of Pisa, Via del Borghetto 80, 56124 Pisa, Italy; Department of Food and Nutritional Sciences, University of Reading, Whiteknights, PO Box 226, Reading, Berkshire RG6 6AP UK

**Keywords:** Auxin, Cytokinin, Ethylene, Floral senescence, Reactive oxygen species, Transcript abundance, Wallflowers

## Abstract

**Background:**

In many species floral senescence is coordinated by ethylene. Endogenous levels rise, and exogenous application accelerates senescence. Furthermore, floral senescence is often associated with increased reactive oxygen species, and is delayed by exogenously applied cytokinin. However, how these processes are linked remains largely unresolved. *Erysimum linifolium* (wallflower) provides an excellent model for understanding these interactions due to its easily staged flowers and close taxonomic relationship to *Arabidopsis*. This has facilitated microarray analysis of gene expression during petal senescence and provided gene markers for following the effects of treatments on different regulatory pathways.

**Results:**

In detached *Erysimum linifolium* (wallflower) flowers ethylene production peaks in open flowers. Furthermore senescence is delayed by treatments with the ethylene signalling inhibitor silver thiosulphate, and accelerated with ethylene released by 2-chloroethylphosphonic acid. Both treatments with exogenous cytokinin, or 6-methyl purine (which is an inhibitor of cytokinin oxidase), delay petal senescence. However, treatment with cytokinin also increases ethylene biosynthesis. Despite the similar effects on senescence, transcript abundance of gene markers is affected differentially by the treatments. A significant rise in transcript abundance of *WLS73* (a putative aminocyclopropanecarboxylate oxidase) was abolished by cytokinin or 6-methyl purine treatments. In contrast, *WFSAG12* transcript (a senescence marker) continued to accumulate significantly, albeit at a reduced rate. Silver thiosulphate suppressed the increase in transcript abundance both of *WFSAG12* and *WLS73*. Activity of reactive oxygen species scavenging enzymes changed during senescence. Treatments that increased cytokinin levels, or inhibited ethylene action, reduced accumulation of hydrogen peroxide. Furthermore, although auxin levels rose with senescence, treatments that delayed early senescence did not affect transcript abundance of *WPS46*, an auxin-induced gene.

**Conclusions:**

A model for the interaction between cytokinins, ethylene, reactive oxygen species and auxin in the regulation of floral senescence in wallflowers is proposed. The combined increase in ethylene and reduction in cytokinin triggers the initiation of senescence and these two plant growth regulators directly or indirectly result in increased reactive oxygen species levels. A fall in conjugated auxin and/or the total auxin pool eventually triggers abscission.

**Electronic supplementary material:**

The online version of this article (doi:10.1186/s12870-016-0766-8) contains supplementary material, which is available to authorized users.

## Background

Petal senescence ends the life of a flower and is an important process for remobilising the nutrient investment to other parts of the plant [1, 2]. Thus, a key feature of petal senescence in many species is its temporal coordination. This ensures that remobilisation has taken place before abscission of the organ. Many of the genes involved in remobilisation during leaf senescence are also up-regulated during petal senescence [3]. However the timing of leaf and petal senescence can be difficult to benchmark. *SENESCENCE ASSOCIATED GENE 12* (*SAG12*) is generally considered as a specific senescence marker both in petals [4] and leaves [5]. However, in wallflower petals this gene was expressed much earlier than it was in leaves that were at a comparable physiological stage of senescence [3]. This suggests that in petals transcript abundance of *SAG12* is an earlier senescence marker.

Several plant growth regulators (PGRs) are implicated in the regulation of petal senescence. However, their interactions with each other, and specific regulatory effects, remain to be fully elucidated. In many species, pollination stimulates the production of ethylene, and ethylene production and sensitivity are primary coordinators of petal senescence [6]. In these species a respiratory burst is accompanied by a sudden rise in ethylene production. This may become autocatalytic by stimulation of ethylene biosynthetic genes [7, 8]. It is also well-documented that treatment of some ethylene sensitive flowers with auxin results in accelerated senescence. This occurs even when detached petals are treated [9, 10]. Ethylene biosynthetic genes are well characterised from flowers of many species [11]. Key regulators of ethylene biosynthesis are aminocyclopropanecarboxylate synthase (ACC synthase or ACS) and aminocyclopropanecarboxylate oxidase (ACC oxidase or ACO). Expression of *ACS* and *ACO* genes is often coordinately regulated during flower senescence (e.g. in *Alstroemeria* [12]; carnation [13] and tomato [14]). In wallflowers, analysis of available ESTs revealed an ACC oxidase-like protein (*WLS73*). Like its *Arabidopsis* homologue [15] *WLS73* is up-regulated during both leaf and petal senescence on microarrays [3].

Auxin induces ethylene production via an increase in the activity of ACC synthase [16]. However, endogenous levels of auxin have only been measured in flowers of a few species [17, 18]. *AUX/IAA* transcripts increase transiently during carnation petal senescence [19] and *Arabidopsis* leaf senescence [20], but are down-regulated in *Arabidopsis* senescing petals [15]. However, a putative orthologue of the *Arabidopsis* auxin-responsive like protein *DFL1* (*DWARF IN LIGHT*) and the tomato *GH3* gene [21], is up-regulated during pollination-induced petunia corolla senescence [22]. The wallflower homologue of this gene, *WPS46* was up-regulated with senescence in both petals and leaves on wallflower microarrays [3]. *DFL1* encodes an IAA-amido-synthase which catalyses indole-3-acetic acid (IAA) conjugation with amino acids, thus modulating the level of free IAA [23].

In contrast to ethylene, cytokinin delays senescence in floral tissues [24]. An inverse relationship between cytokinin content and senescence was established in transgenic petunias over-expressing the isopentenyltransferase (*ipt*) gene driven by the senescence specific *SAG12* promoter. These transgenics had high levels of cytokinin, delayed pollination-induced ethylene production and extended petal lifespan [25]. Treatment with synthetic cytokinin, kinetin or zeatin delayed petal senescence in detached carnation petals and also resulted in a slower rise in ethylene production, possibly through a decrease in ethylene sensitivity [26, 27]. Petal longevity was also increased in carnations by treatment of cut flowers with 6-methyl purine, an inhibitor of cytokinin oxidase [28]. This indicates that cytokinin degradation through cytokinin oxidase may play a significant role in petal senescence. However, the relationship between cytokinin and ethylene appears to be reciprocal since in petunia exogenously applied ethylene induced both petal senescence and inactivation of cytokinins [29].

Reactive oxygen species (ROS) are thought to play an essential role in plant senescence [30]. This is consistent with a loss in antioxidative capacity during the progression of senescence. This loss has been reported in many different species [31, 32]. In petal senescence, the role of ROS remains debatable [33] although a rise in ROS levels accompanies floral senescence in a wide range of both ethylene-sensitive and insensitive species. It is still not known if the increase in ROS levels has a regulatory signalling function or is a consequence of de-regulation of the antioxidant system that occurs as cells enter programmed cell death [24]. An increase in H_2_O_2_ levels was reported upon flower opening in daylily [31], chrysanthemum [34] and rose [35]. In addition, an H_2_O_2_ peak was also observed quite late in petal senescence in tulip after the appearance of key senescence markers such as a rise in proteases and release of cytochrome c from the mitochondria [36]. A similar peak was also reported in daylily, taking place after the sharp rise in ion leakage associated with membrane degradation [31]. In petals, the rise in ROS levels is accompanied by changes in the activity of ROS-related enzymes such as catalase, ascorbate peroxidase and superoxide dismutase and in the levels of antioxidants such as tocopherols [33, 37]. Importantly, these enzymes are all encoded by gene families and changes in activity are linked to changes in the activity of specific isozymes. ROS-responsive genes have been identified in many species. *SAG21* (*At4g02380*) is ROS-inducible in *Arabidopsis* [38, 39] and may play a role in mitigating the effects of ROS on mitochondrial function. However, this gene is also developmentally regulated and responds to other stresses. The wallflower homologue, *WFSAG21* was up-regulated during petal senescence, but not leaf senescence, on wallflower microarrays [3].

Transcriptomic studies have revealed global changes in gene expression during petal senescence. These are mainly related to the activation of catabolism for remobilisation and down-regulation of biosynthesis [15, 24]. Few studies however have examined the effect of treatments that perturb PGR levels and action on the expression of senescence-related genes.

Wallflowers (*Erysimum linifolium*) provide a good model system for studying petal senescence due to their close taxonomic relationship to *Arabidopsis*. This enables easy identification of genes, but provides a much longer lasting flower with a much more predictable developmental programme than the *Arabidopsis* flower [3]. A recent transcriptomic study in wallflowers provided gene markers for different senescence processes, which can be used to study the effects of PGRs on the progression of petal senescence [3]. Here data are presented illustrating the complex relationship between ethylene, cytokinin, auxin and ROS during wallflower petal senescence. We focussed particularly on different methods for delaying senescence and show that treatments that delay senescence also inhibit a rise in ROS. We also show that genes related to different pathways involved in petal senescence including proteolysis, ethylene, auxin and ROS response are differentially regulated when senescence is delayed.

## Results

### Wallflower senescence is ethylene regulated

**Fig. 1:**
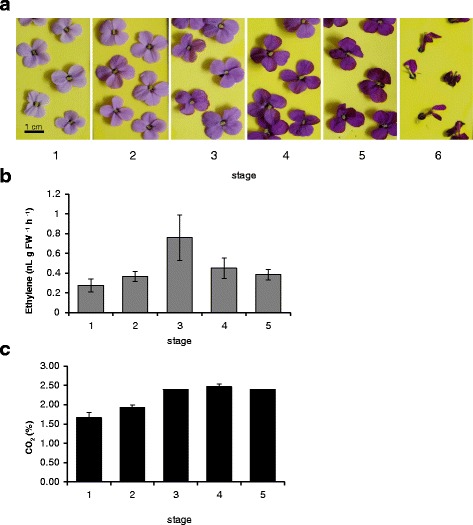
**a** Stages in wallflower senescence: Stage 1 – fully open pale flowers; 4/6 anthers protruding; Stage 2 – larger darker petals, all 6 anthers more visible; Stage 3 – petals held more loosely, beginning to wilt, darker; Stage 4 – petals limp and curled over, darker colour, wilting clearly evident; Stage 5 – clear petal deterioration; Stage 6 - petals beginning to abscise; remaining petals withered (**b**) Ethylene production by isolated petals (**c**) CO_2_ production from isolated wallflowers detected by gas chromatography. Mean (±SE, *n* = 3); stages as in (**a**)

**Fig. 2:**
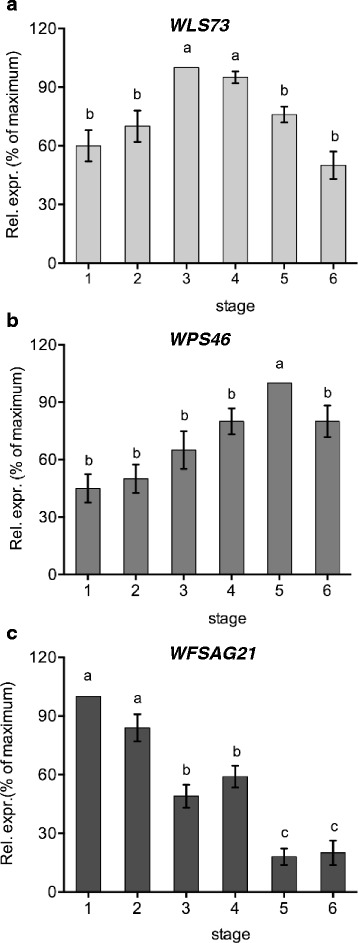
Semi-quantitative RT-PCR analysis of the expression of (**a**) *WLS73*, (**b**) *WPS46* and (**c**) *WFSAG21* genes in different stages of petals (Stage 1 – fully open pale flowers to Stage 6 – abscission) expressed as % of maximum value ± SE (*n* ≥3), normalised to levels of 18S rRNA expression (mean ± SE, *n* = 3; different letters indicate significant differences among stages as determined by one-way ANOVA and a Tukey’s range test)

Ethylene production was analysed in wallflower petals from Stage 1 (first open flower) to flowers showing clear petal deterioration at Stage 5 (Fig. 1a). Ethylene was detectable from the youngest open flower, although the amount produced was quite low (0.28 nL g FW^−1^ h^−1^; Fig. 1b). Ethylene production peaked at Stage 3 (0.76 nL g FW^−1^ h^−1^). A similar pattern was seen for emission from whole flowers (Additional 1: Figure S1). The peak of ethylene production coincided with the maximum CO_2_ emission level, which thereafter remained constant (Fig. 1c). Transcript levels of *WLS73* (a putative ACC oxidase) [40] were relatively low in younger petals but increased significantly (*P* < 0.001) with age (Fig. 2a) reaching a peak at Stage 3–4. This is a similar pattern to the ethylene production.

**Fig. 3:**
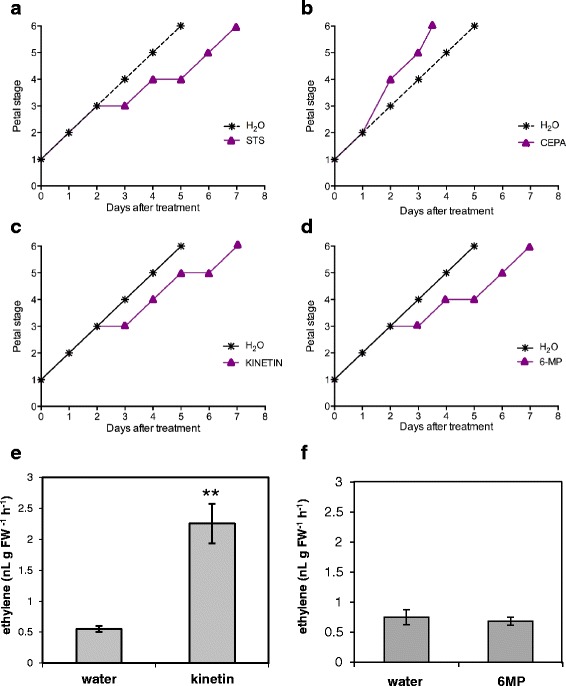
Effect of different treatments on petal senescence and time to abscission (Stage 6) of detached flowers at Stage 1. **a** Treatment with STS consisting of a 1 h pulse (**b**) continuous exposure to CEPA (125 ppm), (**c**) continuous exposure to 0.1 mM kinetin, (**d**) continuous exposure to 0.1 mM 6-methyl purine, (*n* = 10). **e**-**f** Ethylene produced by flowers held for 2 days in either water or 0.1 mM kinetin (**e**), 0.1 mM 6-methyl purine (**f**) (mean ± SE, *n* = 3; in panel **e** ** indicates *P* < 0.01 as determined by a student’s t-test; in panels **a**-**d** no noticeable difference in stage progression was visible between replicates)

Although treatment with silver thiosulphate (STS) delayed time to abscission by 2 days (Fig. 3a) the delay in senescence was not uniform across stages. For the first 2 days of the treatment, senescence progressed at the same rate as controls held in water. However they then remained an extra day at Stage 3 and 4 before resuming senescence at the same rate as the control. Stages were clearly distinct based on changes in petal colour, anther development and petal turgidity and integrity ending with their abscission (Fig. 1a). Floral senescence was also affected by exogenous ethylene generated by 2-chloroethylphosphonic acid (CEPA) (Fig. 3b). This resulted in abscission one and a half days earlier than the water control.

### Kinetin treatment delays senescence and abscission but increases endogenous ethylene production

Treatment of Stage 1 flowers with exogenous kinetin, or inhibition of endogenous cytokinin degradation through treatment with 6-methyl purine (6-MP), an inhibitor of cytokinin oxidase, resulted in a two-day delay in petal abscission. This was due to an extension of Stages 3–5 (Fig. 3c, d).

To determine whether there was a direct effect of kinetin or 6-MP on endogenous ethylene, levels of ethylene accumulation were compared between flowers held in water for 2 days and those treated with kinetin or 6-MP. Surprisingly a more than 3-fold accumulation of ethylene was seen in the kinetin treated flowers (Fig. 3e) despite the delay in senescence elicited by this treatment. In contrast, there was no significant difference in ethylene emission between the 6-MP treated flowers and the control (Fig. 3f).

### Free auxin levels rise, while conjugated auxin levels fall during wallflower senescence

**Fig. 4:**
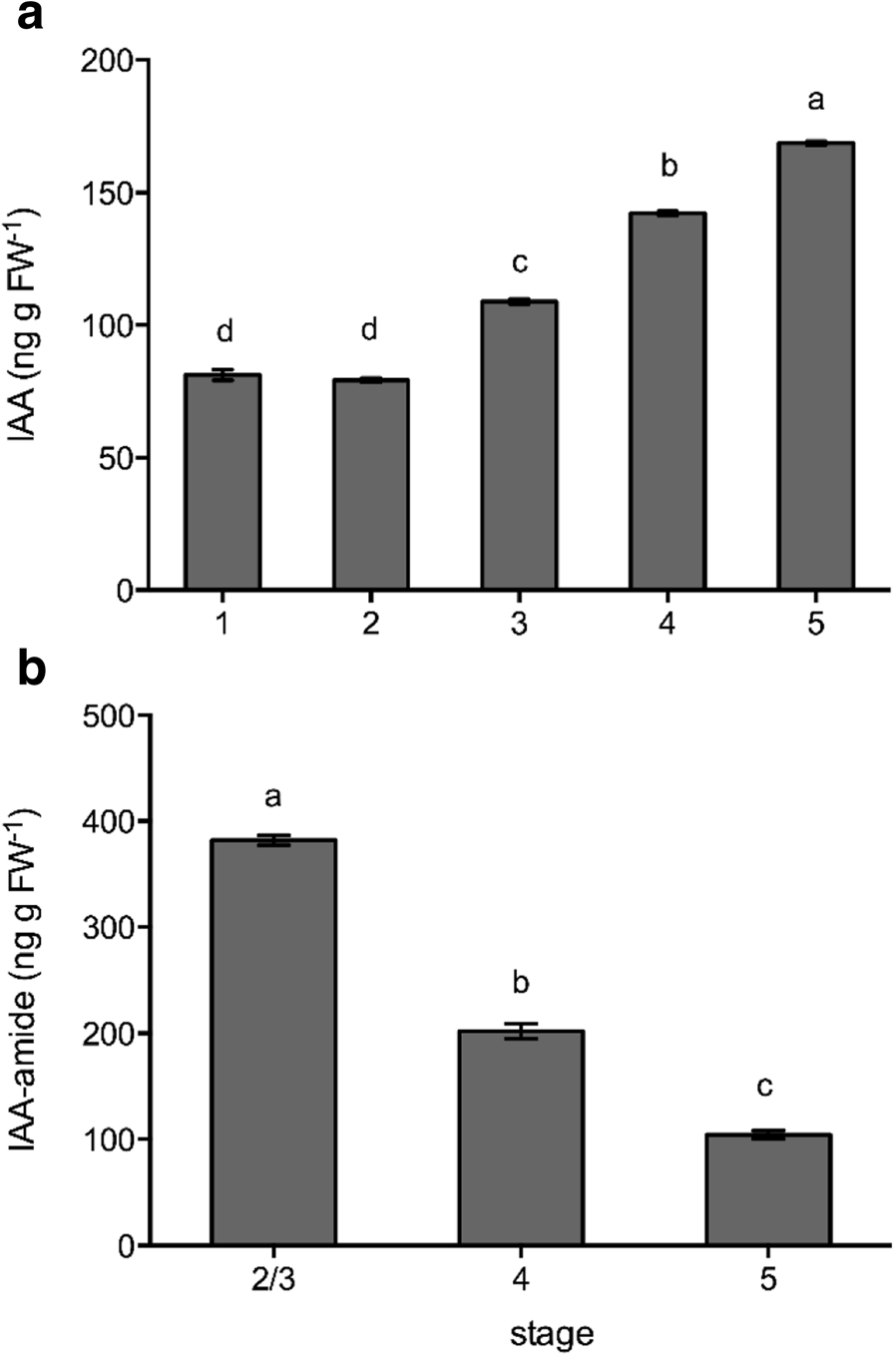
Concentration of free IAA (**a**) and amide-conjugated IAA (**b**) in wallflower petals (Stage 1 – fully open pale flowers to Stage 5 – clear petal deterioration) quantified by GC-MS (mean ± SE, *n* = 3; different letters indicate significant differences among stages as determined by one-way ANOVA and a Tukey’s range test)

Free IAA rose after Stage 2 continuously until Stage 5 (Fig. 4a). IAA-amide fell continuously in open flowers as they senesced from Stages 2/3 to Stage 5 (Fig. 4b). No changes in IAA-ester content were detected during wallflower senescence (data not shown).

Transcript abundance of *WPS46* (an auxin induced gene) in petals remained low during the first two stages of open flowers and was only significantly up-regulated (*P* < 0.001) at Stage 5 (Fig. 2b). This follows the same pattern as endogenous levels of free IAA, but an opposite trend to levels of IAA-amide.

### Reactive oxygen species levels and related enzymes change in activity with senescence

**Fig. 5:**
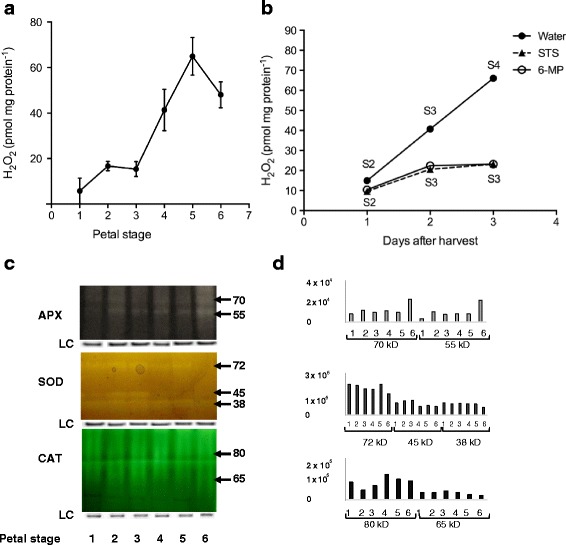
ROS levels and activity of ROS-related enzymes in wallflower petals (**a**) Quantification of relative H_2_O_2_ production in wallflower petals (Stage 1- first fully open flower to Stage 6 – abscission; mean ± SE, *n* = 9); (**b**) Quantification of relative H_2_O_2_ production in detached wallflower petals in the first 3 days following STS and 6-MP treatment. (mean ± SE, *n* = 9). **c** zymograms of APX (ascorbate peroxidase), SOD (superoxide dismutase) and CAT (catalase) isoforms active in wallflower petals at Stages 1–6. Loading control gel images are shown beneath each zymogram; (**d**) quantification of zymogram bands using Image-J (arbitrary units)

Accumulation of ROS in wallflower petals increased with age as measured by H_2_O_2_ concentration (Fig. 5a). There was a significant increase in H_2_O_2_ accumulation between Stage 2 and Stage 4, levelling off thereafter. ROS accumulation was compared in cut flowers harvested at Stage 1 held in water, STS or 6-MP, over a 3 day period. Treatment with 6-MP or STS significantly reduced H_2_O_2_ levels after 2–3 days treatment compared to controls held in water (Fig. 5b).

Analysis of ascorbate peroxidase (APX), superoxide dismutase (SOD) and catalase (CAT) isozyme activities using zymograms revealed complex patterns of changing isozyme activity with petal age (Fig. 5c, d). Two APX isozymes (70 and 55 kDa) were expressed in petals between Stages 1–6. Activities of both isoforms rose between Stage 1 and Stage 2 and then remained fairly constant until a rise at Stage 6. Three SOD isoforms (of 72, 45 and 38 kDa) were detectable with contrasting patterns of expression. All three isoforms declined in senescent petals but activity of the 45 kDa isoform fell much earlier at Stage 4 compared to Stage 6 for the other two isoforms. Two CAT isoforms (of 80 and 65 kDa) were detectable. Activity of the 80 kDal isoform rose between Stage 2 and Stage 4 and then fell back again by Stage 6. Activity of the 65 kDa isoform remained low throughout.

### Perturbation of cytokinin levels or ethylene signalling have different effects on gene expression

**Fig. 6:**
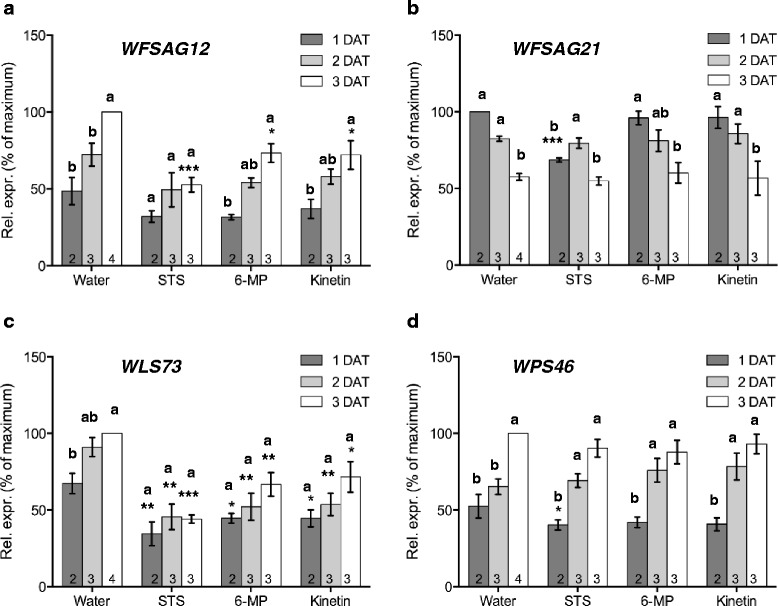
Semi-quantitative RT-PCR analysis of the expression of (**a**) *WFSAG12*, (**b**) *WFSAG21* (**c**) *WLS73* and (**d**) *WPS46* genes in detached flowers treated with water (control), STS, 6-MP and kinetin. Expression is reported as % of maximum value ± SE (*n* ≥3), normalised to levels of 18S rRNA expression. For water-treated flowers days after start of treatment (DAT) 1, 2, and 3 correspond to Stage 2, 3 and 4 respectively. For STS/6MP/kinetin treated flowers, DAT 1, 2, and 3 correspond to Stages 2, 3 and 3 respectively. Asterisks indicate significant differences of relative expression compared to water control on each day as determined by a Dunnett’s test (*P < 0.05; ** *P* < 0.01; *** *P* < 0.001). Letters indicate significant differences within treatments by one-way ANOVA and a Tukey’s range test (*P* < 0.05)

Four genes were selected from the wallflower expressed sequence tags (ESTs) [3] to act as markers for different senescence associated pathways. *WFSAG12* is a senescence-specific cysteine protease [4, 5] and therefore acts as a marker for senescence-associated proteolysis. *WFSAG21* is the wallflower homologue of a ROS responsive-gene in *Arabidopsis* [38, 39]. *WLS73* and *WPS46* act as ethylene and auxin markers respectively. Expression of all four genes was monitored over a 3 day period (days after treatment - DAT 1–3) in cut flowers held in water, or in three different treatments all of which delayed senescence progression (STS, kinetin, and 6-MP). Flowers were harvested at Stage 1 and progressed through to Stage 4 over this time-period when held in water. When subjected to senescence-delaying treatments, flowers only progressed to Stage 3 over this time-period (Fig. 6). In water, transcript abundance of *WFSAG21* fell significantly over days 1–3 after start of the treatment (DAT1 to DAT3), while transcript abundance of *WLS73*, *WPS46* and *WFSAG12* increased significantly. STS treatment suppressed the increase in transcript abundance of *WFSAG12* on DAT3. On DAT3 of STS treatment, *WFSAG12* expression was reduced significantly (*P* < 0.001) compared to the water control (Fig. 6a). Transcript abundance of *WFSAG21* was significantly reduced on DAT1 (*P* < 0.001) by STS treatment compared to the water control, and rose on DAT2 instead of falling (Fig. 6b). Transcript abundance of *WLS73* was affected at all stages by STS treatment, and the rise in its expression from DAT1 to DAT3 was abolished (Fig. 6c). In contrast, transcript abundance of *WPS46* was not affected compared to the water control on each day of treatment. However, all the treatments resulted in a rise on DAT2, which was not seen in the control (Fig. 6d).

## Discussion

### Ethylene and cytokinins

Quantification of ethylene production in wallflower flowers and petals confirmed that ethylene is likely to be an important regulator of petal senescence. This is expected from the close taxonomic relationship of wallflowers to other ethylene-sensitive species such as *Arabidopsis* [41]. In wallflowers, the timing of the peak in endogenous ethylene production was at Stage 3, which is at the very start of visible petal senescence. This ethylene peak coincided with the peak in CO_2_ evolution and suggests that ethylene has a regulatory function in initiating the onset of wallflower petal senescence. The maximum amount of ethylene production was much lower than other ethylene-sensitive flowers such as carnation [8, 42]. In carnation levels of ethylene production are over 20-30-fold higher. However, levels in wallflowers were about 3-fold higher than those reported in *Alstroemeria* [12], which is generally considered ethylene-insensitive. Moreover, the peak in production in wallflowers occurred at a much earlier developmental stage compared to *Alstroemeria*, where ethylene was only detectable just before abscission. This is consistent with a role for ethylene in regulating senescence progression in wallflowers but only abscission in *Alstroemeria* [12]. However, the sensitivity to ethylene of wallflowers was not as high as other flowers e.g. carnation and pelargonium, which are highly sensitive to ethylene. In wallflowers the CEPA treatment accelerated petal senescence by only one and a half days and STS delayed senescence by only 2 days (as previously shown [3]). In carnation and pelargonium ethylene elicits an immediate and dramatic response [6] resulting in severe wilting/abscission within one day of treatment.

Interestingly, the ethylene production pattern exhibited by wallflower petals was also slightly different to other ethylene sensitive flowers [8]. In wallflowers ethylene production was detected at earlier stages than in other ethylene-sensitive species and was not limited to late senescence and abscission. This suggests a role for ethylene in the early phases of wallflower petal development, perhaps during flower opening that occurs just before Stage 1. Previously, ethylene was shown to regulate flower opening in roses [35], perhaps indicating a role for the hormone during cell wall loosening and petal expansion.

As shown previously, [3] STS delayed, and CEPA accelerated, wallflower senescence and abscission when applied to Stage 1 flowers. This contrasts with some ethylene sensitive species such as *Petunia hybrida* and pelargonium [43, 44] where treatment with ethylene on the first day after flower opening is not effective at accelerating senescence. However, effects were slower. At stages when ethylene was effective in petunia, an impact on the rate of senescence progression was seen within 24 h of treatment. In wallflowers, however, progression of senescence was unaltered for the first two days of treatment. These observations support our view that wallflower petals are competent to produce ethylene from an early stage, and this might be associated with flower opening, whilst competence to use ethylene as a signalling molecule to initiate senescence does not occur until later in development.

As previously shown [3], 6-MP treatment had a very similar effect to kinetin application on wallflower senescence. This suggests that inhibiting endogenous cytokinin degradation has a similar effect to providing an external source of the hormone, as was also shown for carnations [28]. Treatment of carnation petals with three different cytokinins: zeatin, kinetin and N^6^-benzyladenine also had very similar effects [27].

In petunia, exogenous ethylene was shown to promote cytokinin inactivation via O-glucosylation and degradation [29]. Conversely, treatment with kinetin in carnation appeared to inhibit ethylene biosynthesis and action [26]. Likewise, increasing endogenous cytokinin levels through expression of the *ipt* gene in petunia resulted in reduced ethylene production [25]. The increase in ethylene accumulation in wallflowers treated with kinetin, at concentrations of the cytokinin that delayed the progression of senescence, was therefore unexpected. Increases in ethylene production were only seen in carnation [26] with very high concentrations of kinetin (>15 μg ml^−1^), which also resulted in a reduced delay in senescence. In wallflowers, the same delay in senescence was obtained with 0.1 mM and 1 mM kinetin (22 and 222 μg ml^−1^; data not shown). This indicates that wallflowers are less sensitive to exogenous kinetin compared to carnation. It also shows that 0.1 mM used in the experiments reported here, is well below toxic levels for this species. However treatment with 6-MP did not result in increased ethylene production in wallflowers. One explanation for these results is that exogenous kinetin treatments make wallflowers less sensitive to endogenous ethylene levels as was previously shown [26]. The kinetin treatment may also disable the mechanism that normally regulates endogenous ethylene concentrations in relation to stage of flower development. In contrast, blocking cytokinin degradation by 6-MP does not affect ethylene production. We may conclude then that the 6-MP does not perturb the ethylene biosynthesis-perception feedback mechanism. This differential effect may be due to different effects on the balance of the endogenous cytokinin pool although the effects may also be indirect.

### Role of auxin

High auxin concentrations have been linked to an inhibition of abscission in non-abscising *Lilium longiflorum* [18]. In *Lilium* both free and conjugated auxins increased continuously peaking in late senescence. Given that petals abscise in wallflowers, it may be significant that although free IAA is high in late petal senescence, conjugated auxins fall by 3-fold during senescence and the total auxin pool falls by 1.7-fold. This suggests that the conjugated auxin levels or the total auxin pool may be the critical factor in triggering abscission. However, effects of exogenous auxin vary between species. In daylilies (*Hemerocallis*), treatment with exogenous auxin delayed senescence [45], while in carnation, exogenous auxin treatments (IAA 5–50 μM) accelerate senescence [9]. In wallflowers, treatments with 13 nM to 52 μM 1-naphthaleneacetic acid (NAA) did not accelerate senescence. However, concentrations above 13 μM caused petal bleaching, suggesting a toxic effect (Additional file 1: Figure S2). Thus auxin seems unlikely to be an early regulator of senescence in wallflowers. This is consistent with the increase in endogenous auxin levels only occurring post-anthesis. Changes in transcript abundance of *WPS46* mirror the rise in free auxin post-anthesis. This is consistent with the expression pattern of *DFL1* [23] and the rice homologoue *GH3*-*8* [46] which are auxin-induced, and the tomato homologue *GH3* whose expression falls with IAA levels [21]. It might be expected that *WPS46* expression would follow the levels of IAA conjugation since rice plants over-expressing the *GH3* homologue had higher levels of conjugated IAA compared to WT [46]. However, the *DFL1* gene acts in auxin signal transduction and its expression in *Arabidopsis* is up-regulated by auxin. Hence its expression in wallflower senescence is consistent with its role in response to changes in free auxin levels. Conversely the level of auxin conjugation is regulated both by conjugation enzymes such as that encoded by *WPS46* but also by biosynthetic enzymes, hence a direct correlation with *WPS46* expression is not expected.

#### Reactive oxygen species

The pattern of change in ROS during wallflower development and senescence is consistent with a sharp increase during petal senescence (Stage 3–5). Both ethylene and ROS levels peak at Stage 3–5 (early senescence) making it difficult to determine whether they regulate each other. Therefore, we tested whether inhibiting senescence either by perturbing ethylene or cytokinin signalling would affect ROS levels. The effect on ROS levels of delaying senescence through inhibiting ethylene signalling (STS) or cytokinin reduction (6-MP) indicates that the ROS is downstream of the PGRs. However, the effect could be direct or indirect through the delay in senescence.

Although *WFSAG21* was selected as a potential ROS-responsive gene, results here indicate that its pattern of expression could be responding to either ethylene or ROS. The timing of the fall in *WFSAG21* suggests a response to ethylene since the fall in *WFSAG21* expression at Stage 5 occurs soon after the fall in ethylene production at Stage 4 while ROS levels only fall later at Stage 6. Responsiveness to both these stimuli occurs in *Arabidopsis*, but it was suggested that *SAG21* was responding primarily to ROS [39]. Results here suggest that, at least in wallflowers, the ethylene response perceived by *WFSAG21* is not mediated by only ROS.

Changes in patterns of some SOD and CAT isoenzyme activities coincided with changes in ROS levels. There was a clear up regulation in the activity of the 80 kDa CAT isozyme and down regulation of the 45 kDa SOD isozyme that coincided with rise in ROS between Stages 3 and 4. APX activity only rose very late in senescence although an up-regulation between Stage 1 and 2 is also visible from the zymograms. The 45 kDa SOD isoform also increased in activity between Stages 1 and 2. CAT levels were very low compared to leaves (data not shown). This suggests that CAT may play a less important role in ROS scavenging during wallflower petal senescence compared to APX and SOD. The activity pattern of some of the wallflower ROS-related isoforms is similar to that in carnations. In carnations both SOD and APX activities peaked in early open flowers which coincided with a rise in ROS, while CAT levels remained constant until late in senescence [47]. Few studies have investigated changes in the activity of different isoforms of ROS related enzymes. However Chakrabarty et al. [34] showed increases in the activity of selected SOD and APX isoforms in chrysanthemum flower heads, which accompanied a steady increase in ROS throughout the stages studied. Note that chrysanthemum flower heads are composed of numerous florets at different stages of development, although the proportion of senescent florets will increase with age. This may account for the lack of clear peaks in ROS or discrete changes in isoenzyme activity with age in chrysanthemum.

### Effects of delaying senescence on transcript abundance

Although inhibition of ethylene signalling with STS, inhibition of cytokinin breakdown with 6-MP or a continuous supply of exogenous cytokinin had similar effects on senescence, effects on transcript abundance of the marker genes was not identical. The increase in *WFSAG12* transcript abundance over time was only suppressed by the STS treatment. *WFSAG12* transcript abundance continued to rise in the 6-MP and kinetin treated flowers, albeit at a reduced rate despite the delay in senescence progression elicited by these two treatments. This suggests that, at least in wallflowers, a rise in *SAG12* transcript abundance may not be as tightly linked to progression in visual signs of senescence in petals as was previously thought. Transcript abundance of *WFSAG21* differed from the water controls only in STS treated flowers where it was significantly lower than controls on day 1. In *Arabidopsis* roots *SAG21* expression is induced both by ethylene and ROS [39]. The increase of *SAG21* transcript abundance elicited by STS treatment adds further weight to the direct effects of ethylene on *WFSAG21* expression.

*WLS73* transcript abundance was reduced by all three senescence-inhibiting treatments at all three time-points. This shows a good correlation between senescence progression and transcript abundance of this gene. Although the pattern of *WLS73* expression followed the rise and fall in ethylene during normal wallflower development and senescence, the increase in endogenous ethylene emission elicited by the kinetin treatment was not accompanied by an increase in *WLS73* transcript abundance. This suggests that the expression of this gene is not solely regulated by ethylene. It also suggests that other ACO family members may be induced by the kinetin treatment to mediate the increased ethylene production. In tomato there are three ACO genes with different temporal and spatial expression in flowers as well as different inducibility e.g. by wounding in leaves [48].

The lack of any significant effect on transcript abundance of *WPS46* by the treatments that delayed senescence adds further support for a lack of involvement of endogenous auxin levels in the regulation of early senescence. Since this gene acts as a marker for auxin responses it also strongly indicates that neither ethylene nor cytokinin treatments affect endogenous auxin levels.

## Conclusions

**Fig. 7:**
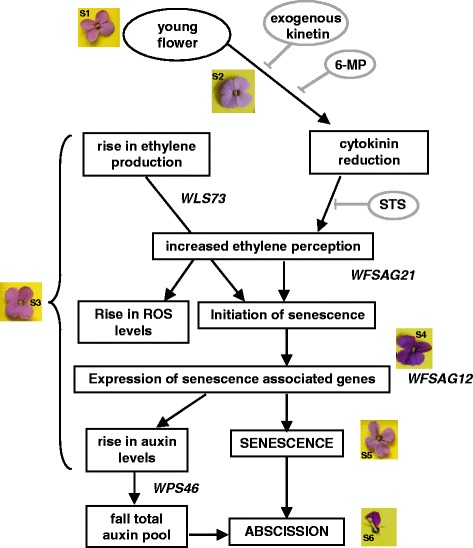
Model outlining the key events relating to plant growth regulators and signals during wallflower senescence. Circled, treatments that affect the progression of senescence

Overall the results can be summarised in a tentative model for pollination-independent senescence in this ethylene-sensitive flower (Fig. 7). As flowers age, endogenous cytokinins fall and ethylene production rises. The fall in cytokinin can be reversed by treatment with 6-MP or supply of exogenous cytokinin. Supply of exogenous cytokinin stimulates ethylene biosynthesis which, however, is seemingly not transduced. Inhibiting cytokinin removal with 6-MP has a similar effect on senescence to cytokinin replacement but without altering ethylene biosynthesis. A combined increase in ethylene perception and reduction in cytokinin triggers the initiation of senescence and these two PGRs directly or indirectly result in increased ROS levels. Once senescence is initiated, a fall in conjugated auxin and/or the total auxin pool eventually triggers abscission.

## Methods

### Plant material

Wallflowers, *Erysimum linifolium* cv. Bowles Mauve obtained commercially from a local garden centre in Cardiff, UK, were grown at the Cardiff University botanical and research garden (Cardiff, UK) either outside or in a greenhouse with temperature set at a minimum of 14 °C. Humidity and photoperiod were not controlled. Petals were collected and staged into seven defined developmental stages according to [3] (see also legend to Fig. 1a) from Stage 1 to Stage 6. For detached flower treatments, flowers were always detached from the plant at Stage 1 (first open flower). Material for protein or RNA extraction was immediately frozen in liquid nitrogen and stored at −80 °C until required.

### Detached flower treatments

Individual flowers were detached from the raceme at Stage 1 (first open flower, often paler in colour than lower flowers) and the pedicel was immediately submerged in distilled water. Flowers were held continuously at 20 °C, 16 h light, 80 μmoles m^−2^ s^−1^ either in distilled water, or in solutions of kinetin (0.1 mM), 6-methyl purine (0.1 mM), NAA (13 nM to 52 μM), or 125 ppm 2-chloroethylphosphonic acid (CEPA; all chemicals from SIGMA-ALDRICH, Dorset, UK). For ethylene inhibitor treatment, flowers were held in STS (4 mM AgNO_3_, 32 mM NaS_2_O_3_) for 1 h and then transferred to water. Concentrations were selected based on those used in published work on other cut flower species [12, 26, 28, 29] and on the results of concentrations ranges previously tested. The CEPA concentration (125 mM) was chosen as 50 mM had no effect on floral longevity, and both 250 mM and 500 mM elicited a very rapid progression from Stage 1 to Stage 4, indicating toxic effects (Additional file 1: Figure S3). The NAA concentration range was based on Shimizu-Yumoto and Ichimura (2010) [49] who used 5 μM NAA in *Eustoma* flowers. For all other treatments the concentration selected was the lowest concentration to affect progression of petal senescence for each chemical without damaging the petals (data not shown as there was no effect). Each experiment consisted of at least ten replicate flowers, monitored daily for senescence stage and day of petal abscission.

### Analysis of endogenous indole-3-acetic acid content

Indolacetic acid (IAA) quantification was carried out according to Mariotti et al. [50]. Approximately 500 mg of petals were used for each Stage and were homogenised in cold 70 % (v/v) acetone (1:5 w/v). To the homogenate, 50 ng of [^13^C_6_] IAA (Olchemim Ltd) were added as an internal standard, then stirred for 4 h at 4 °C. The supernatant was recovered and stored at 4 °C while the pellet was re-extracted twice. The supernatant was reduced to the aqueous phase, adjusted to pH 2.8 and partitioned three times against equal volumes of ethyl ether. The ethyl ether was then evaporated, the dried samples were dissolved in a small volume of 10 % (v/v) aqueous acetonitrile containing 0.1 % (v/v) acetic acid and purified by HPLC.

The aqueous phase after diethyl ether partition was pooled with the extracted pellet and hydrolyzed in 1 N NaOH following the addition of 100 ng of [^13^C_6_] IAA as internal standard. Hydrolysis was continued for 1 h at 27 °C [51] in a capped vial continuously purged with helium. To prevent the conversion of other indolic compounds to IAA under the hydrolysis conditions, a gas purifier (Supelco, Bellefonte, PA) was inserted in the gas line to trap any residual O_2_ [52]. Following hydrolysis, the extracts were centrifuged at 13,000 g for 30 min at 4 °C. The supernatants were acidified to pH = 2.8, and partitioned against diethyl ether as described above. The residual aqueous phases were pooled with the centrifugation pellet and hydrolyzed for 3 h at 100 °C in 7 N NaOH [52], following the addition of 100 ng of [^13^C_6_] IAA as an internal standard. At the end of this hydrolysis, samples were processed as described above.

IAA samples were purified by reverse phase HPLC. The detector was operated at 280 nm wavelength. A C18 Hypersil column (Thermo) 150 × 4.6 mm i.d. particle size 5 μm, eluted at a flow rate of 1 mL min^−1^ was used. Samples were applied to the column and the fraction containing IAA was collected while the column was eluted with a linear gradient of acetonitrile in water and 0.01 % acetic acid, from 10 to 50 % for 15 min and then from 50 to 100 % in 10 min. The fraction was dried under vacuum, and silylated with bis (trimethylsilyl) trifluoroacetamide containing 1 % trimethylchlorosilane (Sigma) for 1 h at 70 °C.

Gas chromatography–mass spectrometry (GC-MS) analysis was performed on a Saturn 2200 quadrupole ion trap mass spectrometer coupled to a CP-3800 gas chromatograph (Varian, Palo Alto, CA) equipped with a MEGA 1 capillary column (MEGA, Legnano, Italy) 25 m × 0.25 mm ID × 0.25 mm film thickness, coated with 100 % dimethylpolysiloxane. For IAA analysis, oven temperature was 120 °C for 2 min, followed by a gradient from 120 °C to 190 °C at 35 °C min^−1^, then from 190 °C to 210 °C at 6 °C min^−1^ and a final ramp from 210 °C to 300 °C at 35 °C min^−1^ with a final hold of 10 min. The following ions were monitored for IAA analysis: m\z 202 and 319 for IAA, and 208 and 325 for the ^13^C-labelled internal standard. Quantification of IAA was carried out by reference to a calibration plot obtained from the GC-MS analysis of a series of mixtures of the standard hormone with its labelled form.

### Analysis of ethylene and CO_2_ emission

For measurement of ethylene emission at different stages of flower development, nine flowers were sealed into a 5 ml collection tube and ethylene was collected for 3 h. Ethylene was measured from the headspace of the tubes using a Hewlett Packard 5890 series 2 Gas Chromatograph (Hewlett Packard, Berks, UK) with a built-in integrator and equipped with a flame ionisation detector set at 150 °C, a 30 m GS-Q column with an internal diameter of 0.53 m and helium carrier gas at a flow rate of 15 ml min^−1^. The column and injector temperatures were both set to 60 °C. A sample injection volume of 100 μl was used and quantified using a 1 μl l^−1^ sample of ethylene as a standard. Headspace in the tubes was analysed for % CO_2_ using an Abiss Print gas analyser, model S/N ABP12WP (EMCO Packaging Systems, Kent, UK). The machine had a resolution of 0.1 %. Three replicates were performed.

Ethylene emission from petal stages was measured from 48 petals which were detached and sealed into a 10 ml collection tube. Ethylene was collected for 12 h and measured on an Agilent 6890 N Network Gas Chromatograph (Agilent Technologies) equipped with a flame ionisation detector set at 150 °C, a 30 m GS-Q column with an internal diameter of 0.32 mm and helium carrier gas at a flow rate of 15 ml min^−1^. Column and injector temperatures were both set to 60 °C. Retention time was confirmed using pure ethylene, and quantification was calibrated using a standard gas mixture (C1-C4 Hydrocarbons, Supelco, 2–3470). Four independent replicates were performed.

For measurement of ethylene evolution following treatments, flowers were detached at Stage 1 and treated for 2 d then three flowers were sealed into a 5 ml collection tube and ethylene collected for 20 h. Headspace concentration of ethylene was measured on a Clarus 500, modified model 2101 analyzer (PerkinElmer, MA, USA), retention time was confirmed using pure ethylene and quantification was calibrated using a standard gas mixture (Scott Specialty Gases, mixture54). Five independent replicates were performed for the kinetin and three for the 6-MP treatment.

### Reactive oxygen species analysis

H_2_O_2_ levels in petals were measured using an Amplex-Red Hydrogen Peroxide/Peroxidase Assay Kit (Molecular probes, Eugene, USA). Petals were ground in liquid nitrogen and then 200 μL of phosphate buffer (20 mM K_2_HPO_4_, pH 6.5) was added to the ground frozen tissue and centrifuged at 13000 rpm, RT, 5 min. After centrifugation, 50 μL of the supernatant was incubated with 100 μM Amplex Red reagent (10-acethyl-3,7-dihydrophenoxazine) and 0.2 units/mL horseradish peroxidase at RT for 30 min in the dark. The fluorescence was quantified using a BIO-TEK FL600 fluorometer (BIO-TEK®) (excitation at 650 nm and emission at 590 nm). The final unit was expressed as pmol mg^−1^ protein.

### Enzyme analyses

Petals were homogenized in 200 μl extraction buffer (100 mM Tris–HCl pH 8.0, 20 % glycerol and 30 mM dithiothreitol (DTT)) with liquid nitrogen at 4 °C. For the analyses of APX activities, the samples were ground with liquid nitrogen in 200 μl extraction buffer 100 mM (potassium phosphate buffer, pH 7.0, containing 5 mM ascorbate and 1 mM EDTA) at 4 °C. Homogenized samples were then centrifuged at 14000 g for 30 min at 4 °C and the resulting supernatant used directly for zymograms, equalising amounts of protein loaded to 10 μg per lane for CAT, 15 μg for SOD and 20 μg for APX zymograms. CAT and APX zymograms were performed according to Zimmermann et al. [53] SOD zymograms were performed according to Orendi et al. [32]. Band intensities on zymograms were quantified using ImageJ software v. 1.47.

### RNA extraction and reverse transcription-PCR

Total RNA isolation from wallflower petals was performed using an RNAqueous® Kit (Ambion, Inc.). DNase treatment was carried out using RNAqueous® Kit (Ambion, Inc.) as described by the manufacturer. cDNA was synthesised using MMLV reverse transcriptase (Promega). For semi-quantitative RT-PCR optimum cycle number was verified by using template dilution of cDNA (100, 50, 25 %) and checking for linear response between template concentration and product abundance at that cycle number and with the specific primers used. A template dilution series was included in each set of PCR reactions per experiment to verify linearity of the amplification at the optimal cycle number. Final values were expressed as percentage of the maximum giving a value of 100 % to highest transcript abundance in each set of reactions for a particular primer pair. At least three reproducible biological replicates were considered for the final transcript abundance quantification. Primers are detailed in Additional file 1: Table S1. PUV primers which bind to 18S rRNA and amplify a 488 bp fragment were used for normalisation as described in [3], and results are presented as a ratio to the 18S rRNA signal.

### Ethics approval and consent to participate

Not applicable.

### Consent for publication

Not applicable.

### Availability of data and materials

Sequences from this article used to design PCR primers are available in the GenBank/EMBL data libraries under accession numbers: *WFSAG21*: AM747887; *WLS73*: AM747819 and *WPS46*: AM747914. All other supporting data are included as additional files.

## Additional file

Additional file 1: Table S1. Primers used for RT-PCR. **Figure S1.** Ethylene production by whole isolated flowers at four developmental stages: stages in wallflower senescence: Stage 1 – fully open pale flowers; 4/6 anthers protruding; Stage 3 – petals held more loosely, beginning to wilt, darker; Stage 4 – petals limp and curled over, darker colour, wilting clearly evident; Stage 5 – clear petal deterioration; **Figure S2.** Effect of different NAA concentrations on progression of petal senescence in detached flowers. Flowers were detached at Stage 1 and treated continuously for 4 days. **Figure S3.** Effect of different CEPA concentrations on progression of petal senescence in detached flowers. (PPTX 356 kb)

## Abbreviations

6-MP: 6-methyl purine
ACC: 1-Aminocyclopropane-1-carboxylic acid
APX: ascorbate peroxidase
CAT: catalase
CEPA: 2-chloroethylphosphonic acid
GC-MS: gas chromatography–mass spectrometry
NAA: 1-naphthaleneacetic acid
SOD: superoxide dismutase
STS: sodium thiosulphate

## References

[1] Rogers HJ (2006). Programmed cell death in floral organs: how and why do flowers die?. Ann Bot.

[2] Rogers HJ (2013). From models to ornamentals: how is flower senescence regulated?. Plant Mol Biol.

[3] Price AM, Aros Orellana DF, Stevens R, Acock R, Buchanan-Wollaston V, Stead AD, Rogers HJ (2008). A comparison of leaf and petal senescence in wallflowers (*Erysimum linifolium*) reveals common and distinct patterns of gene expression and physiology. Plant Physiol.

[4] Macnish AJ, Jiang CZ, Negre-Zakharov F, Reid MS (2010). Physiological and molecular changes during opening and senescence of *Nicotiana mutabilis* flowers. Plant Sci.

[5] Weaver LM, Gan S, Quirino B, Amasino RM (1998). A comparison of the expression patterns of several senescence associated genes in response to stress and hormone treatment. Plant Mol Biol.

[6] Woltering EJ, van Doorn WG (1988). Role of ethylene in senescence of petals morphological and taxonomical relationships. J Exp Bot.

[7] Nichols R (1966). Ethylene production during senescence of flowers. J Hort Sci.

[8] ten Have A, Woltering EJ (1997). Ethylene biosynthetic genes are differentially expressed during carnation (*Dianthus caryophyllus L.*) flower senescence. Plant Mol Biol.

[9] Wulster G, Sacalis J, Janes HW (1982). Senescence in isolated carnation petals. Plant Physiol.

[10] van Staden J (1995). Hormonal control of carnation flower senescence. Acta Hort.

[11] Graham LE, Schippers JHM, Dijkwel PP, Wagstaff C (2012). Ethylene and senescence processes. Ann Plant Rev.

[12] Wagstaff C, Chanasut U, Harren FJ, Laarhoven LJ, Thomas B, Rogers HJ, Stead AD (2005). Ethylene and flower longevity in *Alstroemeria*: relationship between tepal senescence, abscission and ethylene biosynthesis. J Exp Bot.

[13] Savin KW, Baudinette SC, Graham MW, Michael MZ, Nugent GD, Lu CY, Chandler SF, Cornish CE (1995). Antisense ACC Oxidase RNA delays carnation petal senescence. Hort Sci.

[14] Llop-Tous I, Barry CS, Grierson D (2000). Regulation of ethylene biosynthesis in response to pollination in tomato flowers. Plant Physiol.

[15] Wagstaff C, Yang TJ, Stead AD, Buchanan-Wollaston V, Roberts JA (2009). A molecular and structural characterization of senescing *Arabidopsis* siliques and comparison of transcriptional profiles with senescing petals and leaves. Plant J.

[16] Jones ML, Woodson WR (1999). Differential expression of three members of the 1-aminocyclopropane-1-carboxylate synthase gene family in carnation. Plant Physiol.

[17] Arrom L, Munné-Bosch S (2012). Hormonal changes during flower development in floral tissues of *Lilium*. Planta.

[18] Lombardi L, Arrom L, Mariotti L, Battelli R, Picciarelli P, Kille P, Stead AD, Munné-Bosch S, Rogers HJ (2015). Auxin involvement in tepal senescence and abscission in *Lilium*: a tale of two lilies. J Exp Bot.

[19] Hoeberichts FA, van Doorn WG, Vorst O, Hall RD, van Wordragen MF (2007). Sucrose prevents up-regulation of senescence-associated genes in carnation petals. J Exp Bot.

[20] Buchanan-Wollaston V, Page T, Harrison E, Breeze E, Lim PO, Nam HG, Lin FJ, Wu SH, Swidzinski J, Ishizaki K, Leaver CL (2005). Comparative transcriptome analysis reveals significant differences in gene expression and signalling pathways between developmental and dark/starvation-induced senescence in *Arabidopsis*. Plant J.

[21] Mignolli F, Mariotti L, Lombardi L, Vidoz ML, Ceccarelli N, Picciarelli P (2012). Tomato fruit development in the auxin-resistant *dgt* mutant is induced by pollination but not by auxin treatment. J Plant Physiol.

[22] Bai S, Willard B, Chapin LJ, Kinter MT, Francis DM, Stead AD, Jones ML (2010). Proteomic analysis of pollination-induced corolla senescence in petunia. J Exp Bot.

[23] Nakazawa M, Yabe N, Ichikawa T, Yamamoto YY, Yoshizumi T, Hasunuma K, Matsui M (2001). *DFL1*, an auxin-responsive *GH3* gene homologue, negatively regulates shoot cell elongation and lateral root formation, and positively regulates the light response of hypocotyl length. Plant J.

[24] van Doorn WG, Woltering EJ (2008). Physiology and molecular biology of petal senescence. J Exp Bot.

[25] Chang H, Jones ML, Banowetz GM, Clark DG (2003). Overproduction of cytokinins in petunia flowers transformed with *PSAG12-IPT* delays corolla senescence and decreases sensitivity to ethylene. Plant Physiol.

[26] Eisinger W (1977). Role of cytokinins in carnation flower senescence. Plant Physiol.

[27] Mor Y, Spiegelstein H, Halevy AH (1983). Inhibition of ethylene biosynthesis in carnation petals by cytokinin. Plant Physiol.

[28] Taverner EA, Letham DS, Wang J, Cornish E (2000). Inhibition of carnation petal inrolling by growth retardants and cytokinins. Austr J Plant Physiol.

[29] Taverner EA, Letham DS, Wang J, Cornish E, Willcocks DA (1999). Influence of ethylene on cytokinin metabolism in relation to Petunia corolla senescence. Phytochemistry.

[30] Mhamdi A, Queval G, Chaouch S, Vanderauwera S, Van Breusegem F, Noctor G (2010). Catalase function in plants: a focus on *Arabidopsis* mutants as stress-mimic models. J Exp Bot.

[31] Panavas T, Rubinstein B (1998). Oxidative events during programmed cell death of daylily (*Hemerocallis* hybrid) petals. Plant Sci.

[32] Orendi G, Zimmermann P, Baar C, Zentgraf U (2001). Loss of stress-induced expression of *catalase3* during leaf senescence in *Arabidopsis thaliana* is restricted to oxidative stress. Plant Sci.

[33] Rogers HJ (2012). Is there an important role for reactive oxygen species and redox regulation during floral senescence?. Plant Cell Environ.

[34] Chakrabarty D, Chatterjee J, Datta S (2007). Oxidative stress and antioxidant activity as the basis of senescence in chrysanthemum florets. Plant Growth Regul.

[35] Xue J, Li Y, Tan H, Yang F, Ma N, Gao J (2008). Expression of ethylene biosynthetic and receptor genes in rose floral tissues during ethylene-enhanced flower opening. J Exp Bot.

[36] Azad AK, Ishikawa T, Sawa Y, Shibata H (2008). Intracellular energy depletion triggers programmed cell death during petal senescence in tulip. J Exp Bot.

[37] Arrom L, Munné-Bosch S (2010). Tocopherol composition in flower organs of *Lilium* and its variations during natural and artificial senescence. Plant Sci.

[38] Mowla SB, Cuypers A, Driscoll SP, Kiddle G, Thomson J, Foyer CH, Theodoulou FL (2006). Yeast complementation reveals a role for an *Arabidopsis thaliana* late embryogenesis abundant (LEA)-like protein in oxidative stress tolerance. The Plant J.

[39] Mohd Salleh F, Evans K, Goodall B, Machin H, Mowla SB, Mur LAJ, Runions J, Theodoulou FL, Foyer CH, Rogers HJ (2011). A novel function for a redox-related LEA protein (SAG21/AtLEA5) in root development and biotic stress responses. Plant Cell Environ.

[40] Covington MF, Maloof JN, Straume M, Kay SA, Harme SL (2008). Global transcriptome analysis reveals circadian regulation of key pathways in plant growth and development. Gen Biol.

[41] PF Stevens, Angiosperm phylogeny website, version 12, July 2012. http://www.mobot.org/MOBOT/research/APweb/.

[42] Shibuya K, Yoshioka T, Hashiba T, Satoh S (2000). Role of the gynoecium in natural senescence of carnation (*Dianthus caryophyllus* L.) flowers. J Exp Bot.

[43] Whitehead CS, Halevy AH (1989). Ethylene sensitivity: the role of short-chain saturated fatty acids in pollination-induced senescence of *Petunia hybrida* flowers. J Plant Growth Regul.

[44] Deneke CF, Evensen KB, Craig R (1990). Regulation of petal abscission in *Pelargonium × domesticum*. Hort Sci.

[45] Rubinstein B (2000). Regulation of cell death in flower petals. Plant Mol Biol.

[46] Ding X, Cao Y, Huang L, Zhao J, Xu C, Li X, Wang S (2008). Activation of the indole-3-acetic acid–amido synthetase GH3-8 suppresses expansin expression and promotes salicylate- and jasmonate-independent basal immunity in rice. Plant Cell.

[47] Zhang Y, Guo W, Chen S, Han L, Li Z (2007). The role of N-lauroylethanolamine in the regulation of senescence of cut carnations (*Dianthus caryophyllus*). J Plant Physiol.

[48] Barry CS, Blume B, Bouzayen M, Cooper W, Hamilton AJ, Grierson D (1996). Differential expression of the 1-aminocyclopropane-1-carboxylate oxidase gene family of tomato. The Plant J.

[49] Shimizu-Yumoto H, Ichimura K (2010). Combination pulse treatment of 1-naphthaleneacetic acid and aminoethoxyvinylglycine greatly improves postharvest life in cut *Eustoma* flowers. Post Biol Technol.

[50] Mariotti L, Picciarelli P, Lombardi L, Ceccarelli N (2011). Fruit-set and early growth in tomato are associated with increase in IAA, cytokinins and bioactive gibberellins. J Plant Growth Regul.

[51] Nowacki J, Bandurski RS (1980). Myo-Inositol ester of Indole-3-acetic acid as seed auxin precursors of *Zea mays* L. Plant Physiol.

[52] Bialek K, Cohen JD (1989). Quantitation of indoleacetic acid conjugates in bean seeds by direct tissue hydrolysis. Plant Physiol.

[53] Zimmermann P, Heinlein C, Orendi G, Zentgraf U (2006). Senescence-specific regulation of catalases in *Arabidopsis thaliana* (L.) Heynh. Plant Cell Environ.

