# Supplementary material for: Interaction of plant growth regulators and reactive oxygen species to regulate petal senescence in wallflowers (Erysimum linifolium)
Source: BMC Plant Biol. 2016 Apr 2;16:77. doi: 10.1186/s12870-016-0766-8 (PMC4818919; doi:10.1186/s12870-016-0766-8)
Supplement: Additional file 1: Table S1. — Primers used for RT-PCR. Figure S1. Ethylene production by whole isolated flowers at four developmental stages: stages in wallflower senescence: Stage 1 – fully open pale flowers; 4/6 anthers protruding; Stage 3 – petals held more loosely, beginning to wilt, darker; Stage 4 – petals limp and curled over, darker colour, wilting clearly evident; Stage 5 – clear petal deterioration; Figure S2. Effect of different NAA concentrations on progression of petal senescence in detached flowers. Flowers were detached at Stage 1 and treated continuously for 4 days. Figure S3. Effect of different CEPA concentrations on progression of petal senescence in detached flowers. (PPTX 356 kb) [file 12870_2016_766_MOESM1_ESM.pptx]

## Slide 1
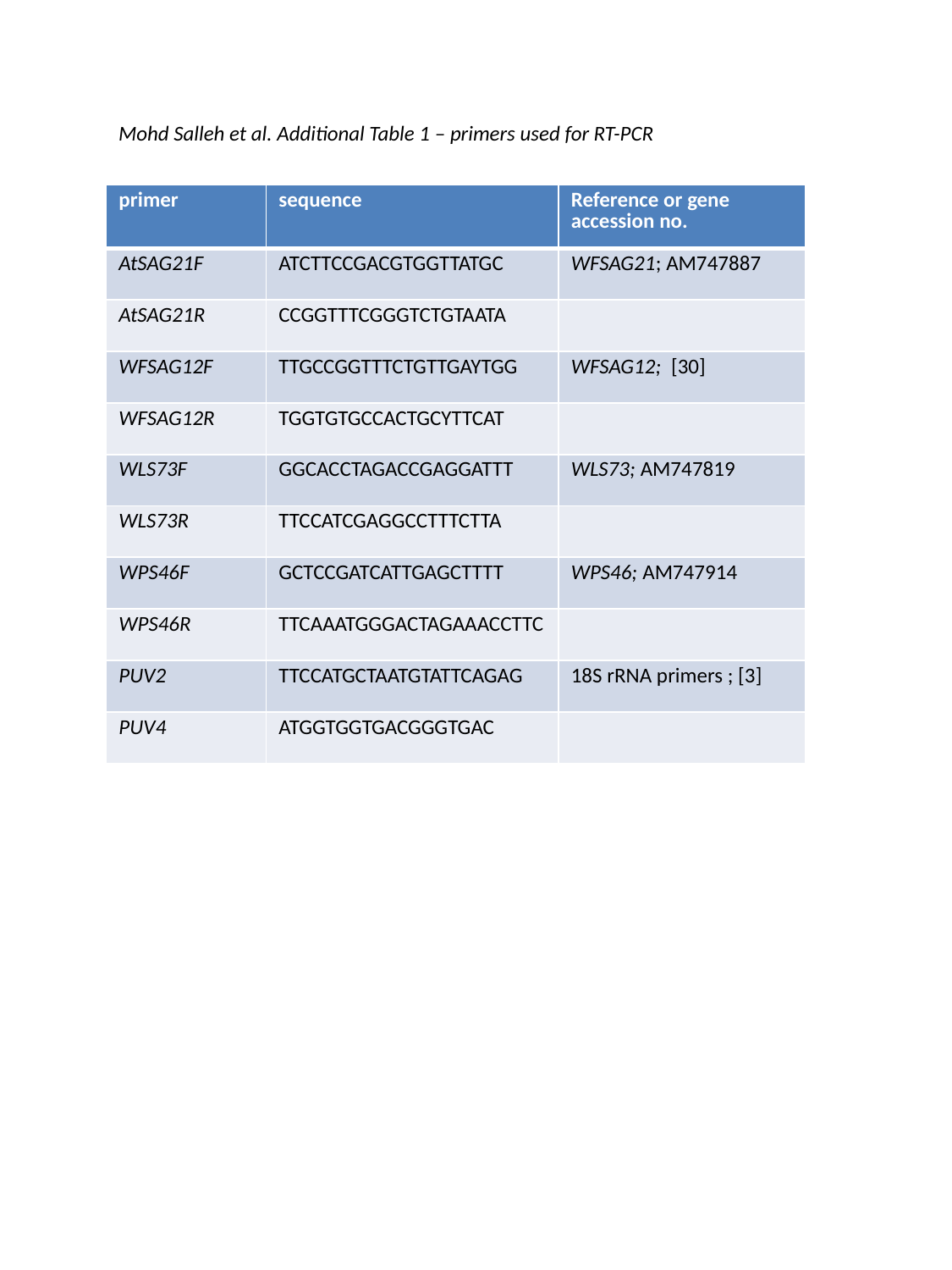

Mohd Salleh et al. Additional Table 1 – primers used for RT-PCR
| primer | sequence | Reference or gene accession no. |
| --- | --- | --- |
| AtSAG21F | ATCTTCCGACGTGGTTATGC | WFSAG21; AM747887 |
| AtSAG21R | CCGGTTTCGGGTCTGTAATA | |
| WFSAG12F | TTGCCGGTTTCTGTTGAYTGG | WFSAG12; [30] |
| WFSAG12R | TGGTGTGCCACTGCYTTCAT | |
| WLS73F | GGCACCTAGACCGAGGATTT | WLS73; AM747819 |
| WLS73R | TTCCATCGAGGCCTTTCTTA | |
| WPS46F | GCTCCGATCATTGAGCTTTT | WPS46; AM747914 |
| WPS46R | TTCAAATGGGACTAGAAACCTTC | |
| PUV2 | TTCCATGCTAATGTATTCAGAG | 18S rRNA primers ; [3] |
| puv4 | ATGGTGGTGACGGGTGAC | |

## Slide 2
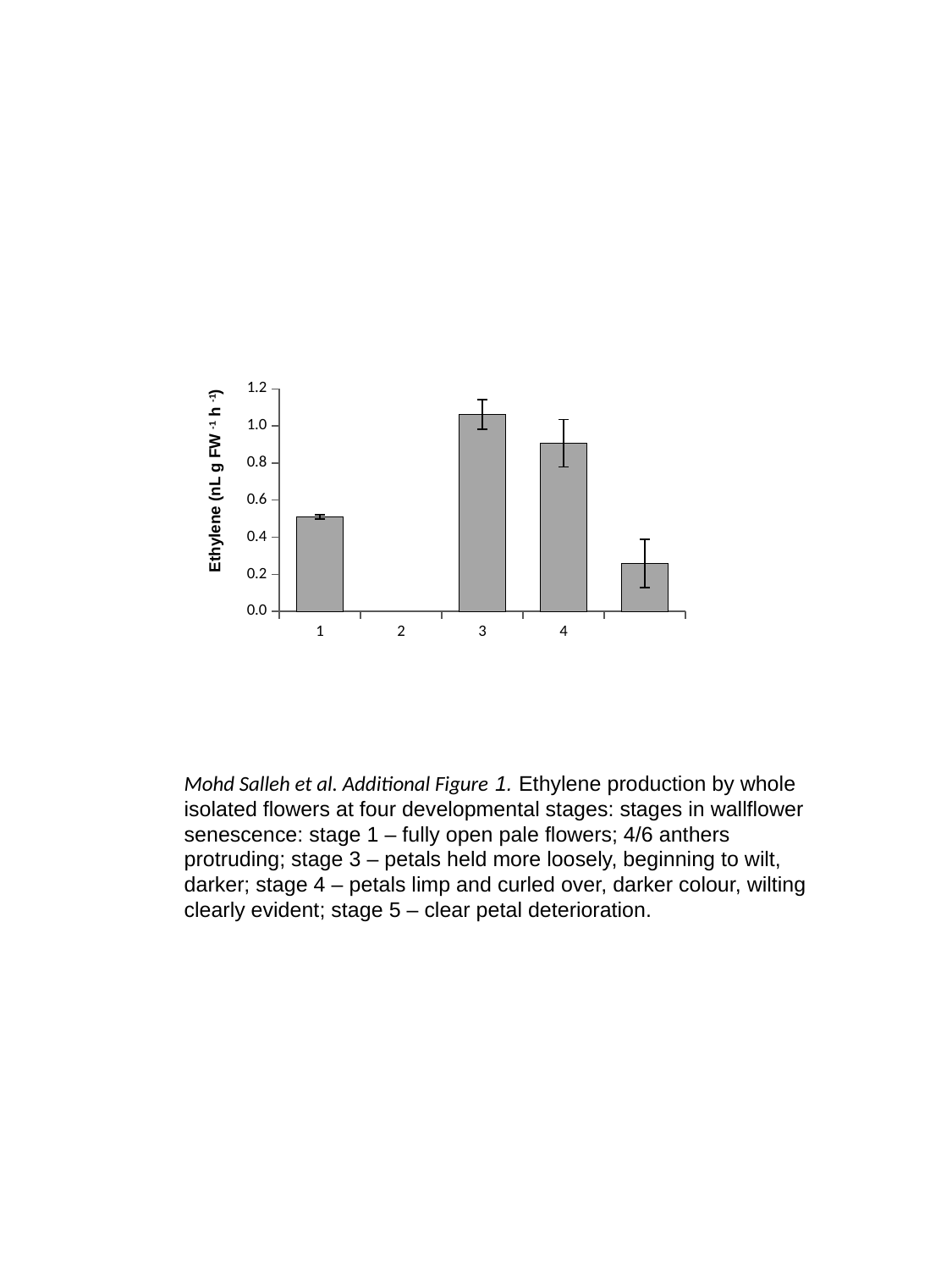

### Chart
| Category | |
|---|---|Ethylene (nL g FW -1 h -1)
Mohd Salleh et al. Additional Figure 1. Ethylene production by whole isolated flowers at four developmental stages: stages in wallflower senescence: stage 1 – fully open pale flowers; 4/6 anthers protruding; stage 3 – petals held more loosely, beginning to wilt, darker; stage 4 – petals limp and curled over, darker colour, wilting clearly evident; stage 5 – clear petal deterioration.

## Slide 3
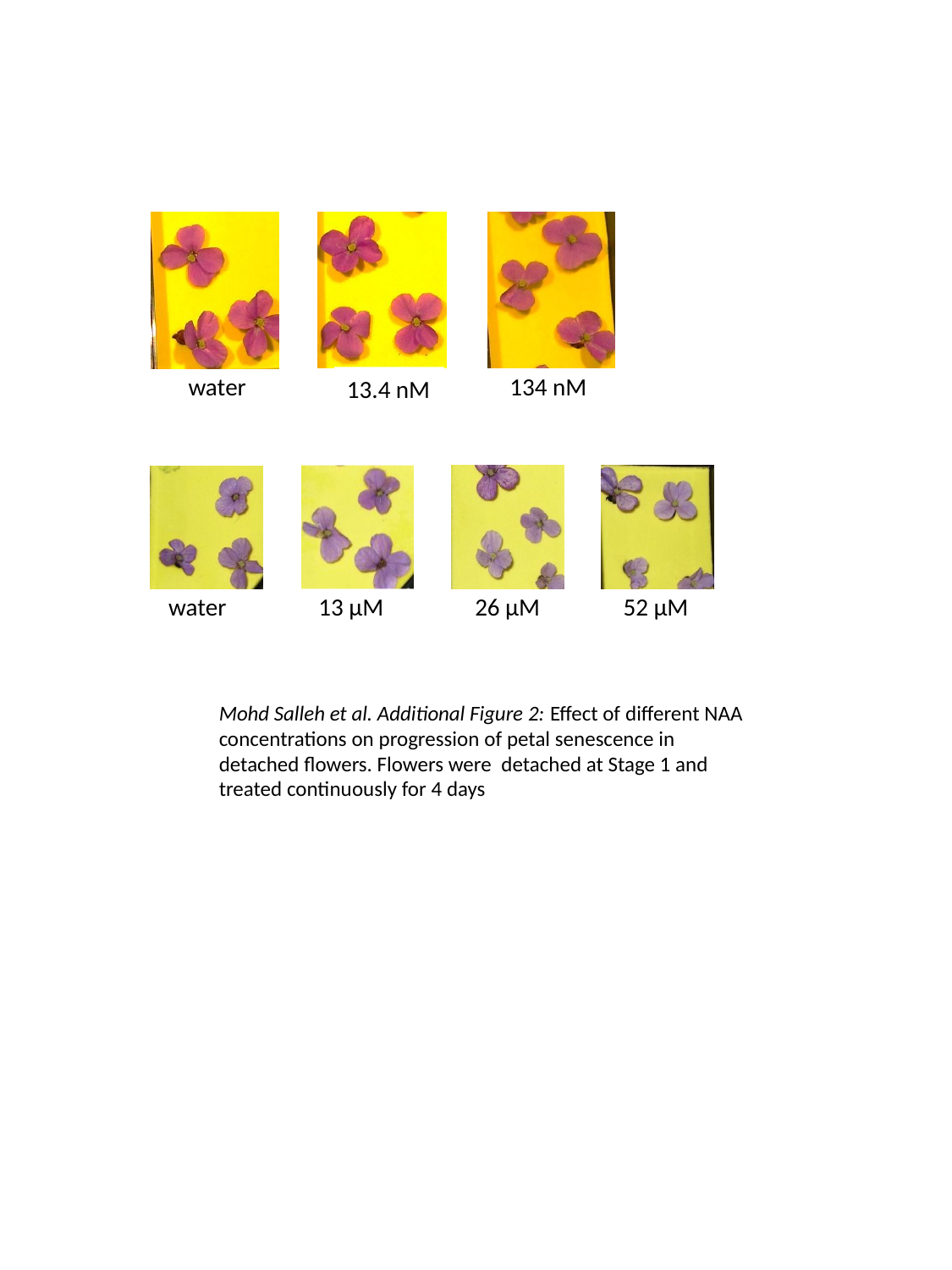

water
134 nM
13.4 nM
13 µM
26 µM
52 µM
water
Mohd Salleh et al. Additional Figure 2: Effect of different NAA concentrations on progression of petal senescence in detached flowers. Flowers were detached at Stage 1 and treated continuously for 4 days

## Slide 4
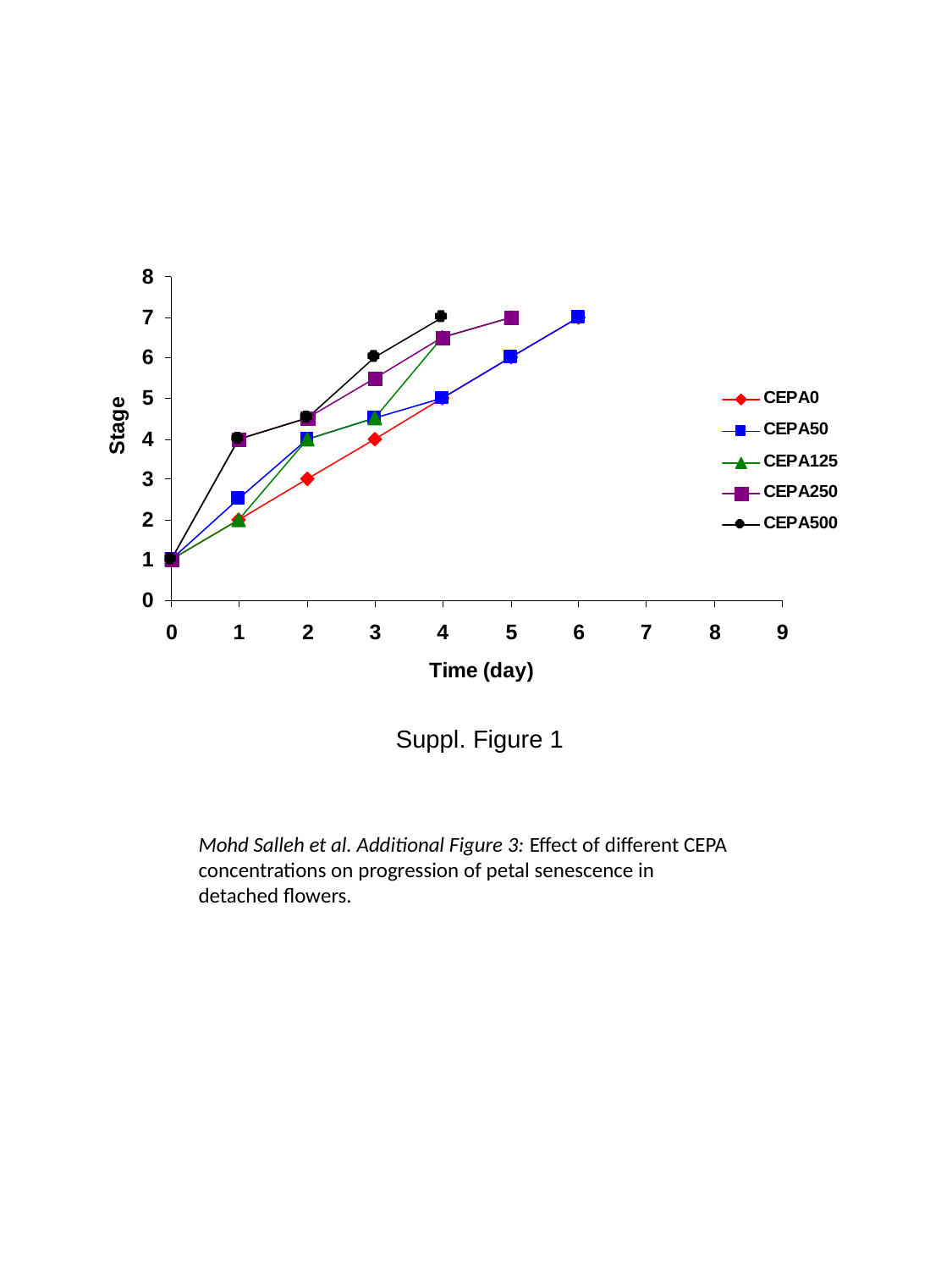

Suppl. Figure 1
Mohd Salleh et al. Additional Figure 3: Effect of different CEPA concentrations on progression of petal senescence in detached flowers.
